# Is it Time to Better Harness Artificial Intelligence for Improving Lifestyle Behaviours?

**DOI:** 10.3389/ijph.2026.1609519

**Published:** 2026-01-27

**Authors:** Jean-Philippe Chaput, Marcus V. V. Lopes, Allana G. LeBlanc, Gary S. Goldfield

**Affiliations:** 1 Children’s Hospital of Eastern Ontario Research Institute, Ottawa, ON, Canada; 2 Department of Pediatrics, University of Ottawa, ON, Ottawa, Canada

**Keywords:** artificial intelligence, behaviour change, digital health, health equity, lifestyle behaviours

## Introduction

Physical inactivity, poor diet, inadequate sleep, and excessive screen time are key modifiable risk factors for chronic disease and mental health problems. These lifestyle behaviours are important contributors to the population burden of obesity, type 2 diabetes, cardiovascular disease, depression, and premature mortality worldwide. Success in achieving sustained population-level improvements in lifestyle behaviours has been rare and often disappointing, despite decades of public health investment in mass media campaigns, health education, and environmental changes. One reason is that lifestyle behaviours are complex by nature, part of daily routines, and influenced by individual, social, and environmental factors. Therefore, we need to adopt new approaches that go beyond traditional strategies if we want to achieve meaningful progress in the future.

Artificial intelligence (AI) is becoming widely used in medicine, healthcare, and consumer technology. However, its potential to improve lifestyle behaviours has only recently drawn attention in public health [1]. Broadly, AI refers to technologies that can learn from data, recognize patterns, and generate predictions or recommendations. AI has the capacity to deliver real-time tailored advice, anticipate lapses in healthy behaviours, and provide timely nudges at a scale that would be impossible through human-led interventions alone.

Many countries continue to face persistent challenges in improving lifestyle behaviours at the population level, and AI may represent a powerful yet underutilized ally in this effort. In this commentary, we explore both the opportunities and the risks of using AI to promote lifestyle behaviour change and reflect on its implications for public health policy and practice.

## The Promise of AI in Lifestyle Behaviour Change

A potential of AI lies in its ability to provide personalization at scale. Public health messages are typically broad and general, but behaviour change depends on many factors such as individual motivation, preferences, and circumstances. AI-driven platforms can integrate passive sensing data from wearables and smartphone sensors with active sensing data from ecological momentary assessments (EMA) to deliver feedback that adapts in real time. For example, a national platform in Singapore applied graph neural networks to generate personalized activity nudges for more than 84,000 participants based on participant characteristics and within-day behaviour fluctuations. Over a 12-week period, these nudges helped increase daily step counts by 6.2% and weekly moderate-to-vigorous physical activity by 7.6% [2].

Another approach with strong potential for lifestyle behaviour modification is reinforcement learning. In a randomized controlled trial of more than 13,000 Fitbit users, participants in a reinforcement learning arm were receiving personalized nudges that updated continuously based on their activity patterns. Compared with groups that received random or fixed messages, people in the reinforcement learning group increased their step counts by 200–300 steps per day after one and two months [3]. These examples illustrate that personalized behavioural coaching driven by AI have the potential to be translated into meaningful health benefits when extrapolated at the population level.

Generative AI (GenAI), often referred to as chatbots, uses large language models to generate predictive responses based on millions of data points. These conversational agents can mimic the dialogue of a health coach and can deliver behavioural support on demand. Two systematic reviews found that AI chatbots have been used successfully to promote smoking cessation, support healthy eating, improve medication adherence, increase physical activity, and enhance sleep, often with high levels of engagement [4, 5]. For many users, the appeal comes from their accessibility, 24/7 availability, and judgment-free interactions. While long-term effectiveness remains uncertain, early evidence suggests that chatbots may offer a scalable and cost-effective way to provide personalized support [6].

AI enables wearable interfaces to turn data into personalized guidance for behaviour change. Consumer-grade smartwatches and smart bands (e.g., Garmin and Apple watches) accurately track steps, heart rate, and sleep; however, they collect so many data points across so many indicators that many users struggle to interpret the data. AI, especially agentic AI, can help by recognizing patterns and predicting when a person deviates from a desired behaviour. Because agentic AI operates from a specified knowledge base, it can provide tailored recommendations and guidance specific to each individual.

In large population samples, providing wearable devices as an intervention has been shown to increase daily step counts by an average of approximately 1,800 steps [7]. AI has the potential to enhance this effect by delivering personalized feedback and coaching, helping users move from simple self-monitoring toward sustained behaviour change.

## The Challenges and Pitfalls

Overall, researchers and health professionals should ensure a human-in-the-loop to prevent errors at the source. This is particularly important when providing guidance that could impact health—for example, recommending physical activity programs without first verifying their suitability for an individual. When using AI to promote healthy lifestyle behaviours, it is also essential to consider potential risks related to health equity, bias, and privacy.

AI tools typically require a smartphone, wearable devices, internet access, and moderate digital and data literacy, which may disadvantage marginalized groups. If unaddressed, these gaps could exacerbate health inequities. Health promotion agencies should explore ways to ensure equitable access, such as providing subsidized devices, public access points, and investments in digital and data literacy.

Unintended (or unknown) bias is another issue to consider in the context of using AI for improving lifestyle behaviours. We know that AI learns from data, and if the data are not representative of the population, recommendations may be misleading or inappropriate. In practice, this means that interventions may work well for those representing the largest tranche of the population (i.e., likely Caucasians in westernized countries), but not for cultural minorities or marginalized groups. It has been reported that community engagement and co-design should be built into every stage of development to prevent unintended harms and optimize efficacy at scale [8].

Privacy, responsible use, and data governance also require attention. Lifestyle data from wearables and apps reveal intimate details of daily life, from sleep patterns to geolocation. Past incidents, such as the exposure of sensitive military locations through aggregated fitness app data [9], highlight the potential risks it may pose. Having strong frameworks for data protection, clear consent processes, and user control over information will be essential for earning and maintaining trust. This also means making sure privacy laws follow ethical principles of transparency and accountability.

Finally, AI should not be mistaken for a replacement for human relationships or social supports. We know that behaviour change is shaped not only by individual motivation but also by economic, social, and environmental factors that no app can address on its own. In mental health, for instance, studies suggest that AI chatbots can help people with mild-to-moderate symptoms but are less effective than human therapy for complex needs [10, 11]. The same lesson applies to lifestyle health. AI should complement, not replace, broader public health strategies or humans.

## Implications for Public Health Practice and Policy

Public health organizations face a crucial task to implement AI systems through strategic planning instead of random adoption. AI tools need to work together with current programs, policies, and environmental support systems in order to establish connections between individualized coaching and available structural resources. To guide this integration, ethical frameworks specific to AI in lifestyle health are needed. Ideally, these frameworks must be co-created by technologists, public health professionals, policymakers, Indigenous leaders, and affected communities. They should emphasize transparency, inclusivity, privacy, and accountability, while embedding Indigenous data governance from the start.

Additionally, cross-sector collaboration will be important. The development of lifestyle health AI applications will require knowledge from computer science, behavioural science, epidemiology, ethics, and community engagement. Partnerships between universities, health agencies, technology companies, and community organizations will also be essential.

Rigorous evaluation will also determine whether AI fulfils its promise. The current state of AI applications shows promise through initial research, but most projects exist only in testing or pilot phases. The evaluation process needs to include randomized controlled trials, real-world implementation studies, and extended follow-up assessments to assess maintenance of behaviour changes over the long-term. Moreover, standardized outcome measures and data-sharing agreements will help build a stronger evidence base. Without these coordinated efforts, there is a risk of investing heavily in technologies that do not deliver meaningful health gains.

### Conclusion

Public health has faced ongoing difficulties when trying to redirect people toward better lifestyle choices, particularly as it relates to improving lifestyle behaviours. AI offers promising capabilities to deliver scalable, personalized, and timely interventions that can drive change at the population level. By addressing issues of equity, bias, privacy, and the risk of overreliance on AI at the expense of other important tools or human judgment, the public health community can help shape AI into an ethical and effective ally for promoting healthier living. It is time for the public health sector to engage proactively with AI. AI systems used correctly can enhance human capabilities while linking people to community resources and delivering personalized support for their daily needs. By seizing this opportunity, the public health community can demonstrate how AI can be harnessed responsibly to improve lifestyle behaviours while maintaining equity, trust, and evidence-based practice.
